# Meetings of Societies

**Published:** 1907-03

**Authors:** 


### MEETINGS OF SOCIETIES.

### Bristol Medico-Chirurgical Society.

*December 12th*, 1906.

Mr. James Taylor, President, in the Chair.

Dr. E. C. Williams showed a **Case of Rheumatoid Arthritis** in a girl aged twelve.

Dr. B. M. H. Rogers showed (1) A **Child of two years of age weighing only seven pounds**, and (2) A case of **Optic Neuritis of sudden onset.**

Dr. Rogers also read notes of a case of **Narcolepsy.**—Dr. A. Rendle Short compared *petit mal* and its persistence to the disease under consideration.—Dr. Stack said the condition suggested that induced by mesmerism, and asked if the pupil had been contracted in the sleep or not. In the mesmeric state he thought the pupil was not contracted, though it was in somnambulism.—Dr. ROGERS replied that the sleep looked like ordinary sleep.

Mr. E. W. HEY GROVES read a paper on **A New Method of Fixation in Excision of the Knee-joint.** The apparatus devised and recommended by the speaker consisted of iron rods, which were passed from side to side through the femur and through the tibia. The projecting ends of these were braced together on each side by two more rods having screw ends. The apparatus fixed the bones immovably together. It was advisable to make a mortise and tenon junction of the femur and tibia. He had used the apparatus successfully in four cases.—Mr. J. LACY FIRTH said the difficulty in fixing the bones in this operation was greater in proportion to the youthfulness of the patient and the softness of the bones, and asked if the speaker's patients had been children or adolescents. He thought that the bones might rotate on the rods passed through them, though he had not actually tested the apparatus, especially if the bones were soft, as was often the case. He had found that steel pins, passed from the tibia into the femoral condyles, crossing one another as they passed from the outer tuberosity of the tibia to the inner condyle, and from the inner tuberosity to the outer condyle, were satisfactory, unless the bones were very soft.—Mr. GROVES replied that his apparatus could be screwed up, so that the femur and tibia were tightly braced together and could not move upon each other. None of the patients had been very young, and three of them twenty years of age or more.

Dr. E. C. WILLIAMS read the concluding notes on a case of **Spleno-megalic Biliary Cirrhosis** (*vide* p. 43). The patient had been shown to the Society a year previously.—Dr. FORTESCUE-BRICKDALE showed the organs obtained at the *post-mortem* examination of the case, and microscopical sections.—Dr. MICHELL CLARKE, from an examination of the specimens, thought the case had not been one of biliary cirrhosis. The specimens looked more like those in ordinary cirrhosis of the liver. It was difficult to deny that the specimens were like those in Banti's disease. Intestinal poisons might cause cirrhosis as well as alcohol.—Dr. WALKER HALL said the microscopical specimens suggested Banti's disease. Some fissures on the right lobe of the liver suggested that the cirrhosis was syphilitic. The sections showed capsular and interlobular cirrhosis.—Dr. CAREY COOMBS thought the changes in the liver were the result of irritation, transmitted to the organ by the bile ducts, systemic arteries, or portal vein. There appeared to be no evidence that the bile ducts or the artery had been the channels of conduction. The cirrhosis was very like the alcoholic form, and the poison had probably come from the intestinal tract through the portal vein.—Dr. WILLIAMS, in reply, said there might be no jaundice in the juvenile type of the disease. By Banti's disease was meant the terminal stage of splenic anæmia of adult type, in which affection the spleen was the first organ to be affected. The child was too young for that, the disease having begun at four years of age. The splenic anæmia of infants is a totally different disease. No results had been observed from anti-syphilitic treatment in the case. Salicylate of soda had done most good.

Dr. KENNETH WILLS read a paper on **Eighty Cases of Lupus Vulgaris treated by Radio-therapy.**

---

*January 9th,* 1907.

Mr. JAMES TAYLOR, President, in the Chair.

Dr. CHARLES read notes on a case of **Multiple Myelomata and Albumosuria.**—Mr. ROGER WILLIAMS pointed out that myelomatosis occurs without albumosuria. Albumosuria was not the disease, it was a secondary phenomenon. Such cases as that described were formerly called multiple sarcomata of bone. It seemed clear that the disease was not sarcomatous, and likely that it belonged to the leucæmic class of diseases, and, like those, was due to an infective agency. The disease had affinities with Paget's osteitis deformans.—Dr. MARKHAM SKERRITT pointed out that not much was known about albumosuria and its causation. It was sometimes met with in pneumonia. He had met with an example of albumosuria in a medical man which persisted for twelve months. Later serum-albumin was found in the urine, with signs of chronic interstitial nephritis. Ultimately the patient died of acute pneumonia.

Dr. MICHELL CLARKE read a paper on **Some Complications of Pneumonia.**—Dr. COOMBS referred to four cases of pneumococcic peritonitis of which he was cognisant. Three of them had been apparently primary. Of these, two children and one adult had recovered. It would be interesting to know whether the affection was more often primary or secondary.—Mr. CARWARDINE mentioned a case of diplococcic peritonitis he had met with. The child developed pneumonia after the peritonitis was established.—Dr. E. C. WILLIAMS mentioned two cases of pneumonia with complications he had treated. In one delusional insanity had arisen. The second was complicated with hiccough, which lasted four or five days, and nearly killed the patient. Obstinate hiccough used to be a more common complication than it was now. In his case morphia had been ineffectual as a remedy.—Dr. MARKHAM SKERRITT mentioned a case of pneumonia in which thrombosis of the pulmonary artery carried off the patient.

Drs. WALKER HALL and CAREY COOMBS demonstrated various macroscopical and microscopical pathological specimens.

*February 13th, 1907.*

Mr. JAMES TAYLOR, President, in the Chair.

An address was given by P. H. PYE-SMITH, Esq., M.D., F.R.C.P., F.R.S., on **Prognosis** (*vide* p. 1).

